# Urogenital schistosomiasis and risk factors of infection in mothers and preschool children in an endemic district in Zimbabwe

**DOI:** 10.1186/s13071-019-3667-5

**Published:** 2019-09-02

**Authors:** Masceline Jenipher Mutsaka-Makuvaza, Zvifadzo Matsena-Zingoni, Agnes Katsidzira, Cremance Tshuma, Nyasha Chin’ombe, Xiao-Nong Zhou, Bonnie Webster, Nicholas Midzi

**Affiliations:** 1 Department of Medical Microbiology, College of Health Sciences, University of Zimbabwe, P. O. Box A178, Avondale, Harare, Zimbabwe; 2 National Institute of Health Research, Ministry of Health and Child Care, P.O. Box CY573, Causeway, Harare, Zimbabwe; 3 Division of Epidemiology and Biostatistics, School of Public Health, Faculty of Health Sciences, University of Witwatersrand, 27 St Andrews’ Road, Parktown, Johannesburg, 2193 South Africa; 4 Harare Central Hospital, P.O Box ST 14, Southerton, Harare, Zimbabwe; 5 Mashonaland Central Provincial Health Office, Ministry of Health and Child Care, Bindura, Mashonaland Central Zimbabwe; 6 National Institute of Parasitic Diseases, Chinese Centre for Disease Control and Prevention, Shanghai, 200025 China; 7 Department of Life Sciences, Natural History Museum, 14 Cromwell Road, London, SW7 5BD UK

**Keywords:** *Schistosoma haematobium*, Prevalence, PSAC, Caregivers, Risk factors, Zimbabwe

## Abstract

**Background:**

To design appropriate schistosomiasis control programmes that include women and preschool-aged children (PSAC) it is essential to assess their disease profile and the risk factors predisposing them to infection. This study aimed to determine the prevalence of urogenital schistosomiasis and the risk factors of infection among PSAC and their caregivers in an endemic area of Zimbabwe.

**Methods:**

A cross-sectional study involving screening for urogenital schistosomiasis infections and treatment of 860 participants [535 children aged ≤ 5 years and 325 caregivers (≥ 15 years)] was carried out in five communities, namely Chihuri, Mupfure, Chakondora, Nduna and Kaziro, in February 2016. Haematuria was recorded for each participant and urine filtration was performed to determine the presence and infection intensity of *Schistosoma haematobium*. A pre-tested questionnaire was administered to the caregivers seeking knowledge, practices and perceptions regarding schistosomiasis. Data analysis was performed using descriptive statistics and logistic regression.

**Results:**

Overall 132 (15.4%) of the 860 participants had *S. haematobium* infections. Among these, 61 (18.7%) of the 325 caregivers and 71 (13.3%) of the 535 children were infected. The infection prevalence was significantly different between caregivers and PSAC (*χ*^2^ = 4.7040, *df *= 1, *P* = 0.030). Children whose caregivers used river water for bathing were more likely to be infected compared to children whose caregivers used protected well water (OR: 2.2, 95% CI: 1.3–3.7). The risks of being infected with schistosomiasis were higher in children whose caregivers were infected compared to children whose caregivers had no infection (AOR: 3.9, 95% CI: 1.7–8.6). In caregivers, those who bathed in river water were at higher risk of schistosomiasis infection compared to those who used water from a protected well (AOR: 3.0, 95% CI: 1.4–6.4).

**Conclusions:**

According to the World Health Organization guidelines, the observed overall prevalence of urogenital schistosomiasis qualifies this area as a moderate risk area requiring mass chemotherapy once every two years. Water contact practices of caregivers, and their perceptions and knowledge regarding schistosomiasis are risk factors for infection in both themselves and PSAC. Thus, disease control efforts targeting caregivers or PSAC should include health education and provision of alternative clean and safe water sources.

## Background

Schistosomiasis is a major public health problem in many tropical and sub-tropical regions [1–5]. The disease is endemic in 78 countries worldwide where approximately 206.4 million people require preventive chemotherapy, with 92% of them residing in Africa [3]. In 2010, the national survey in Zimbabwe reported an overall prevalence of 22.7% for *S. haematobium* and *S. mansoni* infections in school-aged children (SAC) [6]. Although *S. haematobium* is the most prevalent in the country, the distribution of both species is dependent on the availability and abundance of their intermediate host snails, *Bulinus globosus* and *Biomphalaria pfeifferi*, respectively [6, 7]. The presence of suitable intermediate hosts and transmission of schistosomiasis is also dependent on favourable climatic factors with temperature being the main driving factor [4, 8]. Other factors contributing to the distribution of the disease include water contact practice, lack of access to safe water and low socio-economic status [9–11]. Thus, profiling these factors in endemic communities is vital for improving control programmes towards achieving maximum benefit. Schistosomiasis control in sub-Saharan Africa has been implemented mainly through preventative chemotherapy involving the mass drug administration (MDA) of praziquantel, primarily to SAC [5, 6, 10, 12, 13]. However, the most recent World Health Assembly resolution of 2012 (WHA 65.21) [14] on schistosomiasis elimination advocates for the inclusion of all high-risk groups including preschool-aged children (PSAC) (1–5 years-old) and adults into the MDA campaigns [5, 15–23]. While it is acknowledged that most PSAC are too young to engage in water contact activities, there is a plethora of evidence showing that young pre-schoolers are regularly exposed to schistosome infection by their caregivers who take them to fresh water sources or use water from these sources for bathing. The older and more mobile group of PSAC visit water bodies for swimming, washing, bathing and other activities [22, 24]. However, there is a paucity of data on knowledge, perceptions, water contact and sanitary practices of women and the risk factors predisposing their PSAC and themselves to schistosomiasis infection in endemic areas. Thus, it is imperative to elucidate the epidemiological determinants of childhood and maternal schistosomiasis risk and infection.

Although the burden and morbidity, due to schistosomiasis, in women and PSAC has been separately described in Zimbabwe [15, 16, 19], this study aimed to determine the distribution of urogenital schistosomiasis infection in PSAC and their caregivers in Shamva District, the most endemic district in the country [6]. The study also aimed to determine if the caregivers’ water contact practices, knowledge and perceptions are risk factors predisposing them and PSAC to schistosomiasis.

## Methods

### Study area

**Fig. 1:**
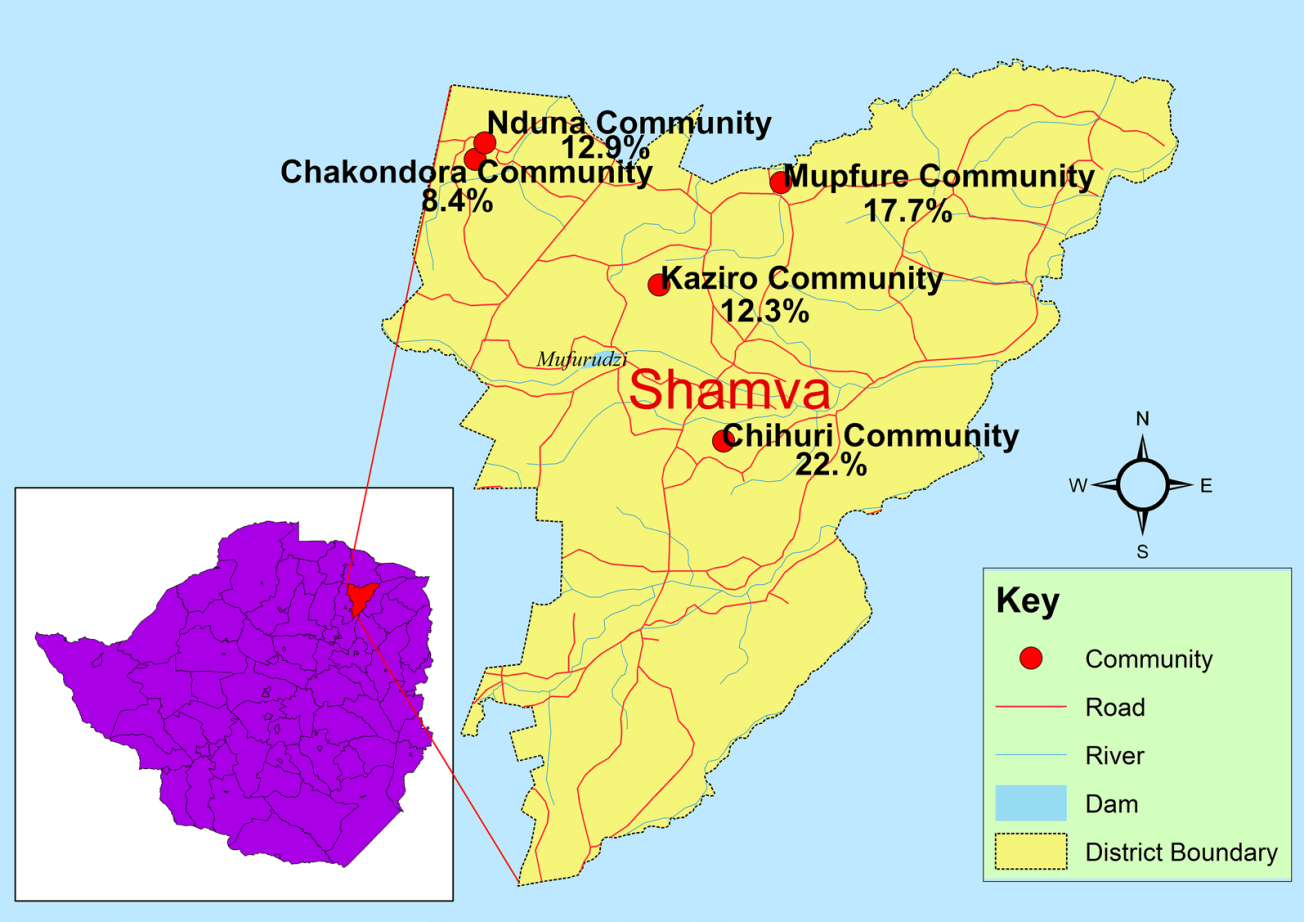
Map showing study area and prevalence of urogenital schistosomiasis for each community. The combined schistosomiasis prevalence for caregivers and children in each community is shown

The study area has been previously described [15]. The study was conducted in five communities (Mupfure, Chihuri, Nduna, Chakondora and Kaziro) located in Shamva District, Mashonaland Central Province, Zimbabwe (Fig. 1). Shamva District was purposively selected due to the high prevalence (62%) of schistosomiasis among SAC recorded during the 2010 national epidemiological survey [6].

Most inhabitants of the area are of the Shona ethnic group, mostly Christians. The study communities are located approximately 55 km from Bindura, the nearest town with a provincial hospital. Within the study area, there is one inadequately staffed clinic with two nurses but no doctors. Some communities are as far as 10 km away from the clinic. The study communities are located near one big river (Mupfure), and three small rivers (Nyamaruru, Nyarukunda and Kamoyo) serving as sources of fresh water for most household activities and farming purposes. The area receives high rainfall, averaging 175 mm/month during the rainy season (November–March) but is dry between May and October. Most communities do not have a safe water supply for drinking and only one borehole is located within a 5 km radius. The study communities are heavily involved in vegetable gardening and tobacco farming, which serves as the main source of income for a significant number of families.

### Study design

The study was cross-sectional in design and was conducted within a major study investigating host-schistosome interactions: Disease burden in children aged five years and below, mothers and compliance during a one-year schistosomiasis control programme in a district described as highly endemic in Zimbabwe.

### Inclusion and exclusion criteria

**Fig. 2:**
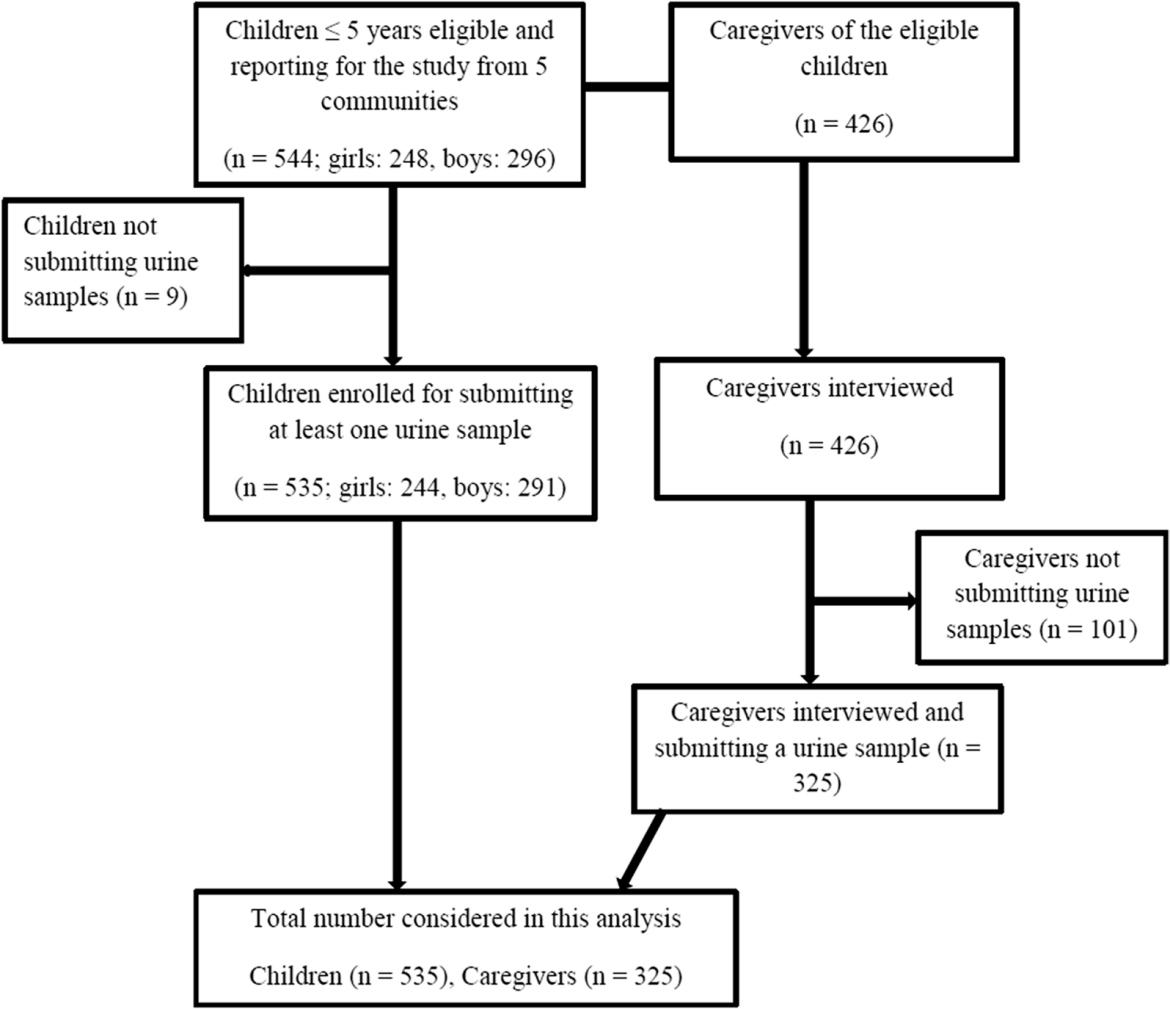
Description of participant recruitment into the study. Children were recruited first and all caregivers of eligible children were included into the study

Figure 2 illustrates how participants were recruited and included into the study. Children aged 5 years and below and their caregivers (17–49 years) were recruited into the study. There were few exceptions in which non-targeted elderly women > 49 years and young adult women below 17 years were included in the study if they were guardians of the PSAC. The PSAC were included in the study if they managed to submit a urine sample to test for urogenital schistosomiasis infection. A total of 426 caregivers and 544 children were eligible for enrolment into the study. The difference in number of caregivers *vs* PSAC enrolled was the result of some caregivers bringing more than one child aged 5 years and below. Among the 544 eligible children, 9 could not provide a urine sample and so did not participate in the study. All the 426 caregivers responded to the knowledge, perceptions and practices (KPP) questionnaire, of which 325 also managed to submit urine samples for urogenital schistosomiasis diagnosis.

### Sample size

In estimating the sample size of the study population, the 62% schistosomiasis prevalence rate previously reported was used [6]. The assumption that the prevalence of schistosomiasis in SAC is a proxy of the community prevalence was also considered [25]. The required sample size of 363 PSAC and 363 caregivers was calculated using Dobson’s formula as follows:

$$ n = \frac{{{Z^{2}}_{{{\alpha \mathord{\left/  \right. \kern-2pt} _2}}} pq}}{{d^{2} }} $$

where *Z* is the *Z*-value for the 95% confidence interval, that is alpha = 5% (*Z* = 1.96), *p* is the proportion/ prevalence of the outcome to be investigated (*p* = 0.62), *q* = 1 − *p* = 0.38, *d* is the precision for the given confidence interval expected expressed as a decimal (*d* = 0.05) and *n* = 363

The final sample size contained 535 children and 325 caregivers, giving a total of 860 participants.

### Sampling procedures

Caregivers and their PSAC were invited to a sampling centre through the community nurse and village health workers. For each community, the study used the Expanded Programme for Immunisation centre (e.g. primary health centre or school) as a sampling centre. Each participant was given a unique identification number. For the caregivers, the same identification number was used on the questionnaire, urine sample and the parasitological results of the participant. For the children, the same identification number was used on the urine sample and parasitological results of the participant. In addition, the child’s study identification number was also captured on the caregiver’s questionnaire.

### Questionnaire

To assess the risk factors of urogenital schistosomiasis in the study population, a KPP questionnaire was initially developed in English and translated into the local language (Shona). A separate person blinded to the original English version back-translated the Shona version to English. The validated Shona survey questionnaire was used to collect data from the caregivers. Trained village health workers and nurses administered the questionnaire, which recorded the participants’ demographic characteristics, and knowledge, perceptions and practices related to schistosomiasis infection using both close ended and open ended questions to capture elements of qualitative and quantitative data. Additional information on the knowledge of current and past occurrences of urogenital schistosomiasis, symptoms, prevention methods of infection, water contact practices and risk group perceptions were captured from the caregivers.

### Specimen collection for parasitological diagnosis

Individual participants (caregivers and their PSAC) were provided with screw-capped urine containers, for each individual, labelled with their identification number. Urine was collected from children on two consecutive days. For logistical and compliance reasons, only one urine sample was collected from caregivers. Approximately 50 ml of urine was collected from each participant between 10:00 and 14:00 h, a period when peak egg excretion is expected [26]. For the PSAC, their caregivers were directed on how to assist the children in collecting the urine sample. Urine bags (Hollister 7511 U-Bag Urine Specimen Collector, Hollister Inc., Chicago, IL, USA) labelled with the child’s identification number were provided to caregivers with children who could not provide urine samples in screw cap containers. The caregivers were instructed on how to use the urine bags. The collected samples were transported to the field laboratory for processing and analysis within 2 h.

### Urine laboratory analysis and microscopic examination

Collected urine samples were first examined macroscopically for visible blood (haematuria). Secondly, a urinary dipstick reagent strip (Spinreact, Girona, Spain) was used for the detection of microhaematuria following the manufacturer’s instructions. The urinalysis reagent strip was dipped into the sample bottle containing urine and immediately removed. The resulting change in colour of the reagent strip was recorded after 1 min and this was compared with the manufacturer’s chart to estimate the amount of blood in the urine sample. The concentration of blood in urine was semi-quantitatively recorded following the manufacturer’s guidelines as: negative (−); light intensity (+); medium intensity (++); and heavy intensity (+++). Blood in urine was considered to indicate *S. haematobium* infection. Caregivers were requested to indicate if they were menstruating. For all menstruating women, blood in urine was not considered to indicate *S. haematobium* infection.

Lastly, the urine samples were examined for *S. haematobium* eggs using the urine filtration technique as described by Mott et al. [27]. In brief, 10 ml of each thoroughly mixed urine sample was syringed through a Nytrel filter (12 µm pore size and 13 mm in diameter). The filter was then placed on the ordinary microscope slide and a drop of 10% Lugol’s iodine was added. The filter membrane was examined under a microscope for *S. haematobium* eggs. The intensity was expressed as the number of eggs per 10 ml of urine (EP10 ml). Participants were considered infected if at least one egg was detected in any of their urine samples.

### Administration of praziquantel to study participants

Following parasitological diagnosis of all urine samples, participants found positive for urogenital schistosomiasis (those with eggs in urine) were given bread and orange juice to eat, after which they were administered a single dose of praziquantel at the recommended dose of 40 mg/kg body weight by the local nurse. For the PSAC, those found positive were given praziquantel crushed and dissolved in orange juice for easy swallowing of the estimated praziquantel dose.

### Data analysis

Data were double-entered in Microsoft Excel and exported to STATA v.15.2.0 (Stata Corp, College Station, TX, USA) for analysis. Age was categorized into the following groups: < 1, 1, 2, 3, 4, 5 years for the children, and 15–19, 20–24, 25–29, 30–34, 35–39, 40–44, 45–49, ≥ 50 years for the caregivers. Infection intensity for each infected individual was categorized according to the World Health Organization guidelines (1–49 eggs/10 ml, light intensity; ≥ 50 eggs/10 ml, heavy intensity) [25]. Mann-Whitney test was used to test the difference in *Schistosoma* egg intensity, whilst association between categorical variables was assessed using a Chi-square test. Continuous variables that were non-normal were summarised using median and interquartile range.

Descriptive analysis of the KPP data was performed and results were reported as frequencies and percentages for all categorical variables. To determine the risk factors of schistosomiasis infection, we performed both univariate and multiple variable logistic regression analysis to identify if *S. haematobium* infection in either children or caregivers was associated with KPP information. Odds ratios (ORs) and 95% confidence intervals (95% CI) were estimated and reported. A significance level was set at 5%.

## Results

### Demographic characteristics of participants

In total, 860 participants [535 (62.2%) PSAC, 325 (37.8%) caregivers] were recruited from the five communities as follows: Chakondora, 190 (22.1%); Chihuri, 184 (21.4%); Kaziro, 130 (15.1%); Mupfure, 255 (29.7%); and Nduna, 101 (11.7%) (Table 1). Of the total participants, 569 (66.2%) were female participants (caregivers = 325 and PSAC = 244) while 291 (33.8%) were male PSAC participants. The mean age (SD) of caregivers in the study population was 32.3 (9.0) years. In the PSAC group, the mean (SD) age of the children was 3.0 (1.2) years. There was no significant difference in mean age between males and females in the PSAC group (*t*_(533)_ = − 1.3037, *P* = 0.1929).

**Table 1:** Demographic characteristics and prevalence of *S. haematobium* among PSAC and caregivers

Variable	No. examined (%)	No. infected (prevalence)	95% CI	*χ*^2^-value	*P*-value
Overall	860 (100)	132 (15.4)	13.0–17.9		
Community					
Chakondora	190 (22.1)	16 (8.4)	4.9–13.3	17.375	0.002
Chihuri	184 (21.4)	42 (22.8)	17.0–29.6		
Kaziro	130 (15.1)	16 (12.3)	7.2–19.2		
Mupfure	255 (29.7)	45 (17.7)	13.2–22.9		
Nduna	101 (11.7)	13 (12.9)	7.0–21.0		
Study group					
Caregivers	325 (37.8)	61 (18.7)	14.7–23.4	4.704	0.030
Children	535 (62.2)	71 (13.3)	10.5–16.4		
Gender					
Male	291 (33.8)	36 (12.4)	8.8–16.7	1.946	0.163
Female	569 (66.2)	96 (16.9)	13.9–20.2		
Age categories (years)					
Children				17.203	0.004
< 1	7 (1.3)	0 (0)	–		
1	76 (14.2)	4(5.3)	1.5– 12.9		
2	80 (15.0)	10 (12.5)	6.2– 21.8		
3	171 (32.0)	17 (9.9)	5.9–15.4		
4	129 (24.1)	22 (17.1)	11.0–24.7		
5	72 (13.5)	18 (25.0)	15.3–36.6		
Adults				5.546	0.594
15–19	9 (2.8)	3 (33.3)	7.5–7.0		
20–24	54 (16.6)	15 (27.8)	16.5–41.6		
25–29	65 (20.0)	12 (18.5)	9.9–3.0		
30–34	82 (25.2)	13 (15.9)	8.7–25.6		
35–39	67 (20.6)	10 (14.9)	7.4–25.7		
40–44	27 (8.3)	5 (18.5)	6.3–38.1		
45–49	6 (1.9)	1 (16.7)	0.4–64.1		
≥ 50	15 (4.6)	2 (13.3)	1.7–40.5		

*Note*: Female includes both female children and adult women

### Prevalence of *S. haematobium* in caregivers and PSAC

The overall prevalence of *S. haematobium* in our study was 15.4% (95% CI: 13.0–17.9) (Table 1). *Schistosoma haematobium* infection was significantly higher in caregivers (61; 18.7%) than in PSAC (71; 13.3%) (*χ*^2^ = 4.7040, *df* = 1, *P* = 0.030). The prevalence of infection was also significantly different among all the study participants age groups (*χ*^2^ = 26.4519, *df* = 13, *P* = 0.015). Likewise, *S. haematobium* prevalence was significantly different among communities (*χ*^2^ = 17.3750, *df* = 4, *P* = 0.002), being most prevalent in Chihuri (22.8%) and least prevalent in Chakondora (8.4%) (Fig. 1, Table 1). However, no difference was observed in urogenital schistosomiasis prevalence after stratification by gender (*χ*^2^ = 3.0015, *df* = 1, *P* = 0.083). In the PSAC group, *S. haematobium* prevalence significantly differed among age groups (*χ*^2^ = 17.2035, *df* = 5, *P* = 0.004). Notably, a prevalence of 0% was recorded in the < 1-year group, while 25% was recorded among those aged 5 years (Table 1).

### *Schistosoma haematobium* infection intensity

A total of 132 *S. haematobium*-positive participants had a median egg intensity of 5.2 (interquartile range: 2–11.3) eggs per 10 ml of urine. Table 2 describes the infection intensity among participants. The proportion of light infection intensities (93.2%) was significantly higher than heavy infections (6.8%) (*Z* = 14.0324, *df* *=* 1, *P* ≤ 0.001). This significant difference was also reflected among the communities (*χ*^2^ = 10.8532, *df* = 4, *P* = 0.028). Caregivers had significantly higher light infection intensities (93.4%) than heavy infections (6.6%) (*Z* = − 9.5968, *P* ≤ 0.001). However, no significant difference existed between the caregivers’ infection intensities between the communities (*χ*^2^ = 4.2680, *df* = 4, *P* = 0.371). The PSAC had significantly higher light intensity infections (93.0%) than heavy infections (7.0%) (*Z* = − 10.2380, *P* ≤ 0.001). Similarly, infection intensity in the PSAC was significantly different among communities (*χ*^2^ = 12.5339, *df* = 4, *P* = 0.014). However, no significant difference was observed when PSAC were classified in relation to the gender (*χ*^2^ = 0.1860, *df* = 1, *P* = 0.666).

**Table 2:** Infection intensity estimates among PSAC and caregivers

Variable	Total				Caregivers				PSAC			
	No. examined	Infection intensity			No. examined	Infection intensity			No. examined	Infection intensity		
		Light *n* (%)	Heavy *n* (%)	*P*-value		Light *n* (%)	Heavy *n* (%)	*P*-value		Light *n* (%)	Heavy *n* (%)	*P*-value
Total infected	132	123 (93.2)	9 (6.8)	< 0.001	61	57 (93.4)	4 (6.6)	< 0.001	71	66 (93.0)	5 (7.0)	< 0.001
Sex												
Male	36	33 (91.7)	3 (8.3)	0.672					36	33 (91.7)	3 (8.3)	0.666
Female	96	90 (93.8)	6 (6.3)		61	57 (93.4)	4 (6.6)		35	33 (94.3)	2 (5.7)	
Community												
Chakondora	16	16 (100)	0 (0)	0.028	9	9 (100)	0 (0)	0.371	7	7 (100)	0 (0)	0.014
Chihuri	42	39 (92.9)	3 (7.1)		21	19 (90.5)	2 (9.5)		21	20 (95.2)	1 (4.76)	
Kaziro	16	12 (75.0)	4 (25.0)		12	10 (83.3)	2 (16.7)		4	2 (50.0)	2 (50.0)	
Mupfure	45	43 (95.6)	2 (4.4)		13	13 (100)	0 (0)		32	30 (93.8)	2 (6.3)	
Nduna	13	13 (100)	0 (0)		6	6 (100)	0 (0)		7	7 (100)	0 (0)	

### Macrohaematuria, microhaematuria and microhaematuria intensity

Macrohaematuria was examined in 834 participants. Overall, macrohaematuria was observed in 14 (1.7%) participants. There was no significant difference between the prevalence of macrohaematuria in caregivers (8; 2.5%) compared to PSAC (6; 1.2%) (*χ*^2^ = 1.9775, *df* = 1, *P* = 0.160). However, there was a significant difference in the prevalence of macrohaematuria between the communities (*χ*^2^ = 18.8894, *df* = 4, *P* = 0.001).

Overall, microhaematuria was detected in 10.9% of the 832 participants. There was marginal evidence of difference in the microhaematuria between caregivers (13.5%) and PSAC (9.3%) (*χ*^2^ = 3.7039, *df* = 1, *P* = 0.054). The prevalence of microhaematuria was also significantly different between communities (*χ*^2^ = 13.1950, *df* = 4, *P* = 0.010). Microhaematuria intensity was insignificantly higher in caregivers than in PSAC (*χ*^2^ = 4.3226, *df* = 3, *P* = 0.229) (Table 3).

**Table 3:** Prevalence and intensity of microhaematuria and macrohaematuria among participants

Variable	Total			Caregiver			Children			*P*-value
	No. examined	Prevalence *n* (%)	95% CI	No. examined	Prevalence *n* (%)	95% CI	No. examined	Prevalence *n* (%)	95%CI	
Macrohaematuria										
Overall	834	14 (1.7)	0.1–2.8	325	8 (2.5)	1.1–4.8	509	6 (1.2)	0.4–2.5	0.160
Community										
Chakondora	183	2 (1.1)	0.1–3.9	52	1 (1.9)	0.0–10.3	131	1 (0.8)	0.0–4.2	0.001
Chihuri	182	1 (0.6)	0.0–3.0	81	0 (0)	–	101	1 (1.0)	0.0–5.4	
Kaziro	130	8 (6.2)	2.7–11.8	63	6 (9.5)	3.6–19.6	67	2 (3.0)	0.4–10.4	
Mupfure	248	2 (0.8)	0.0–2.9	86	1 (1.2)	0.0–6.3	162	1 (0.6)	0.0–3.4	
Nduna	91	1 (1.1)	0.0–6.0	43	0 (0)	–	48	1 (2.1)	0.0–11.1	
Microhaematuria										
Overall	832	91 (10.9)	8.9–13.3	325	44 (13.5)	10.0–17.7	507	47 (9.3)	6.9–12.1	0.054
Community										
Chakondora	183	10 (5.5)	2.7–9.8	325	6 (11.5)	4.5–23.4	507	4 (3.1)	0.8–7.6	0.010
Chihuri	180	27 (15.0)	10.1–21.1	325	12 (14.8)	7.9–24.4	507	15 (15.2)	8.7–23.8	
Kaziro	130	18 (13.9)	8.4–21.0	325	13 (20.6)	11.5–32.7	507	5 (7.5)	2.5–16.6	
Mupfure	248	31 (12.5)	8.7–17.3	325	10 (11.6)	5.7–20.3	507	21 (13.0)	8.2–19.1	
Nduna	91	5 (5.5)	1.8–12.4	325	3 (7.0)	1.5–19.1	507	2 (4.2)	0.5–14.3	
Microhaematuria intensity										
+	832	30 (3.6)	2.4–5.1	325	13 (4.0)	2.1–6.7	507	17 (3.4)	2.0–5.3	0.229
++	832	34 (4.1)	2.8–5.7	325	18 (5.5)	3.3–8.6	507	16 (3.2)	1.8–5.1	
+++	832	27 (3.3)	2.1–4.7	325	13 (4.0)	2.1–6.7	507	14 (2.8)	1.5–4.6	

### Caregivers’ knowledge of schistosomiasis

Table 4 describes the knowledge of the study population regarding schistosomiasis symptoms, treatment, prevention and control. However, the difference in knowledge of haematuria as a symptom of schistosomiasis infection was not significantly different (*χ*^2^ = 0.7634, *df* = 1, *P* = 0.382) between the infected (38, 66.7%) and uninfected caregivers (179, 72.5%). All infected caregivers knew that schistosomiasis could be treated and that treatment can be obtained at a health centre. Treatment was considered to prevent and control the disease by 70 (23.0%) women. The difference in knowledge of provision of safe water, sanitation and hygiene (WASH) facilities would also prevent schistosomiasis infection and transmission between the infected and the uninfected caregivers was significant (*χ*^2^ = 3.9261, *df* = 1, *P* = 0.048).

**Table 4:** Association of caregivers’ knowledge regarding schistosomiasis with infection status among themselves and their children

Category^a^	Children^b^	Child’s infection status			Caregivers	Caregiver’s infection status		
	Total	Infected	Uninfected	*P*-value	Total	Infected	Uninfected	*P*-value
Had knowledge of schistosomiasis	502 (93.8)	69 (97.2)	433 (93.3)	0.208	304 (93.5)	57 (18.8)	247 (81.3)	0.973
Mother’s history of infection								
Had infection before	251 (50.0)	41 (59.4)	210 (48.5)	0.092	148 (48.7)	24 (42.1)	124 (50.2)	0.270
Caregivers indicating they are currently infected								
Yes	152 (30.3)	38 (55.1)	114 (26.3)	< 0.001*	96 (31.6)	21 (36.8)	75 (30.4)	0.537
No	177 (35.3)	13 (18.8)	164 (37.9)		102 (33.6)	16 (28.1)	86 (34.8)	
I don’t know	173 (34.5)	18 (26.1)	155 (35.8)		106 (34.9)	20 (35.1)	86 (34.8)	
Schistosomiasis symptoms								
Haematuria	364 (72.5)	52 (75.4)	312 (72.1)	0.568	217 (71.4)	38 (66.7)	179 (72.5)	0.382
Weight loss	132 (26.3)	15 (21.7)	117 (27.0)	0.355	73 (24.0)	12 (21.1)	61 (24.7)	0.562
General body weakness	64 (12.8)	6 (8.7)	58 (13.4)	0.277	40 (13.2)	10 (17.5)	30 (12.2)	0.277
Headache	6 (1.2)	1 (1.5)	5 (1.2)	0.834	5 (1.6)	0 (0)	5 (2.0)	0.279
Nausea	43 (8.6)	9 (13.0)	34 (7.9)	0.152	27 (8.9)	7 (12.3)	20 (8.1)	0.317
Dysuria	45 (9.0)	7 (10.1)	38 (8.8)	0.712	32 (10.5)	8 (14.0)	24 (9.7)	0.338
Poor school performance	7 (1.4)	1 (1.5)	6 (1.4)	0.967	5 (1.6)	0 (0)	5 (2.0)	0.279
Abdominal pain	34 (6.8)	7 (10.1)	27 (6.2)	0.230	24 (7.9)	3 (5.3)	21 (8.5)	0.414
Genital itchiness in women	12 (2.4)	2 (2.9)	10 (2.3)	0.768	8 (2.6)	2 (3.5)	6 (2.4)	0.650
Infertility	7 (1.4)	2 (2.9)	5 (1.2)	0.251	8 (2.6)	1 (1.8)	7 (2.8)	0.646
Recurrent illness	11 (2.2)	2 (2.9)	9 (2.1)	0.666	6 (2.0)	1 (1.8)	5 (2.0)	0.895
I don’t know	21 (4.2)	3 (4.4)	18 (4.2)	0.941	18 (5.9)	2 (3.5)	16 (6.5)	0.392
Can schistosomiasis be treated? (Yes)	501 (99.8)	69 (100)	432 (99.8)	0.689	303 (99.7)	57 (100)	246 (99.6)	0.630
Knowledge of Health Centre as a place for schistosomiasis treatment	499 (99.4)	68 (98.6)	431 (99.5)	0.323	301 (99.0)	57 (100)	244 (98.8)	0.403
Prevention and control^c^								
Treatment using anti-schistosomal medicine	139 (27.7)	18 (26.1)	121 (27.9)	0.749	70 (23.0)	13 (22.8)	57 (23.1)	0.965
Avoiding contact with unprotected water bodies	220 (43.8)	25 (36.2)	195 (45.0)	0.171	135 (44.4)	27 (47.4)	108 (43.7)	0.618
Health education	43 (8.6)	10 (14.5)	33 (7.6)	0.059**	32 (10.6)	8 (14.0)	24 (9.8)	0.344
Provision of WASH^d^ facilities	70 (14.0)	9 (13.0)	61 (14.1)	0.811	54 (17.8)	5 (8.8)	49 (19.9)	0.048*

^a^For the variables with a yes or no response, only the number (%) of the yes responses are documented

^b^Children were not interviewed but they are described against their caregiver’s responses

^c^More than one response was considered

^d^Water, sanitation and hygiene

*Significance level of 0.05; **Borderline significance of 0.05

### Caregivers’ perceptions and practices regarding schistosomiasis

Perceptions and practices of caregivers in relation to schistosomiasis are described in Table 5. Regarding the current national schistosomiasis treatment programme, for those who knew about schistosomiasis, 303 (99.7%) caregivers, including all infected participants, indicated that the programme is important.

**Table 5:** Association of caregivers’ perceptions and practices regarding schistosomiasis with infection status among themselves and their children

Category^a^	Children^b^	Child’s infection status			Caregivers	Caregiver’s infection status		
	Total	Infected	Uninfected	*P*-value	Total	Infected	Uninfected	*P*-value
Perceptions								
Is the current national schistosomiasis treatment programme important? (Yes)	501 (99.8)	69 (100)	432 (99.8)	0.689	303 (99.7)	57 (100)	246 (99.6)	0.630
Which group is most likely infected?^c^								
Children aged ≤ 5 years	242 (48.1)	34 (49.3)	208 (48.0)	0.848	152 (50.0)	21 (36.8)	131 (53.0)	0.028*
School-aged children	387 (77.1)	50 (72.5)	337 (77.8)	0.325	226 (74.3)	47 (82.5)	179 (72.5)	0.120
Adult women	97 (19.3)	19 (27.5)	78 (18.0)	0.063	56 (18.4)	9 (15.8)	47 (19.0)	0.570
Adult men	64 (12.8)	13 (18.8)	51 (11.8)	0.102	39 (12.8)	3 (5.3)	36 (14.6)	0.058**
Girls	76 (15.1)	12 (17.4)	64 (14.8)	0.574	45 (14.8)	6 (10.5)	39 (15.8)	0.313
Boys	86 (17.1)	14 (20.3)	72 (16.6)	0.453	49 (16.1)	8 (14.0)	41 (16.6)	0.635
Practices								
Domestic source of water								
Borehole	337 (63.0)	34 (47.9)	303 (65.3)	0.005*	207 (63.7)	32 (52.5)	175 (66.3)	0.043*
Dam	13 (2.4)	2 (2.8)	11 (2.4)	0.820	6 (1.9)	0 (0)	6 (2.3)	0.235
River	110 (20.6)	27 (38.0)	83 (17.9)	< 0.001*	68 (20.9)	12 (19.7)	56 (21.2)	0.790
Well	153 (28.6)	24 (33.8)	129 (27.8)	0.297	98 (30.2)	23(37.7)	75 (28.4)	0.154
Place of doing laundry								
River	395 (73.8)	57 (80.3)	338 (72.8)	0.670	232 (71.4)	42 (68.9)	190 (72.0)	0.518
Dam	32 (6.0)	2 (2.8)	30 (6.5)		21 (6.5)	4 (6.6)	17 (6.4)	
Garden well	20 (3.7)	2 (2.8)	18 (3.9)		12 (3.7)	1 (1.6)	11 (4.2)	
Borehole	15 (2.8)	2 (2.8)	13 (2.8)		11 (3.4)	4 (6.6)	7 (2.7)	
Well at home	73 (13.6)	8 (11.3)	65 (14.0)		49 (15.1)	10 (16.4)	39 (14.8)	
Laundry time								
Morning	306 (57.2)	39 (54.9)	267 (57.5)	0.204	180 (55.4)	37 (60.7)	143 (54.2)	0.651
Afternoon	191 (35.7)	30 (42.3)	161 (34.7)		122(37.5)	20 (32.8)	102 (38. 6)	
Late afternoon	38 (7.1)	2(2.8)	36 (7.8)		23 (7.1)	4 (6.6)	19 (7.2)	
Bathing place								
River	199 (37.2)	37 (52.1)	162 (34.9)	0.034*	114 (35.1)	29 (47.5)	85 (32.2)	0.123
Dam	5 (0.93)	1 (1.4)	4 (0.9)		5 (1.5)	0 (0)	5 (1.9)	
Garden well	7 (1.3)	2 (2.8)	5 (1.1)		6 (1.9)	2 (3.3)	4 (1.5)	
Borehole	18 (3.4)	2 (2.8)	16 (3.5)		11 (3.4)	2 (3.3)	9 (3.4)	
Protected well	306 (57.2)	29 (40.9)	277 (59.7)		189 (58.2)	28 (45.9)	161 (61.0)	
Bathing time								
Morning	28 (5.2)	3 (4.2)	25 (5.4)	0.452	17 (5.2)	2 (3.3)	15 (5.7)	0.174
Afternoon	110 (20.6)	11 (15.5)	99 (21.3)		73 (22.5)	9 (14.8)	64 (24.2)	
Late afternoon	397 (74.2)	57 (80.3)	340 (73.3)		235 (72.3)	50 (82.0)	185 (70.1)	
Child goes with mother to the river for laundry								
Yes	362 (67.7)	60 (84.5)	302 (65.1)	0.003*	213 (65.5)	42 (68.9)	171 (64.8)	0.478
No	143 (26.7)	11 (15.5)	132 (28.5)		90 (27.7)	17 (27.9)	73 (27.7)	
Sometimes	30 (5.6)	0 (0)	30 (6.5)		22 (6.8)	2 (3.3)	20 (7.6)	
Where does child play when mother is doing laundry?								
Playing in shallow water	226 (42.2)	41 (57.8)	185 (39.9)	0.016*	150 (46.2)	32 (52.5)	118 (44.8)	0.198
Playing in a water filled dish outside the river	81 (15.1)	11 (15.5)	70 (15.1)		44 (13.5)	8 (13.1)	36 (13.6)	
Outside the water	168 (31.4)	16 (22.5)	152 (32.8)		92 (28.3)	11 (18.0)	81 (30.7)	
I don’t go with my child to the river or dam	60 (11.2)	3 (4.2)	57 (12.3)		39 (12.0)	10 (16.4)	29 (11.0)	
Child goes with the mother to the river for bathing								
Yes	272 (50.8)	52 (73.2)	220 (47.4)	< 0.001*	163 (50.2)	33 (54.1)	130 (49.24)	0.687
No	211 (39.4)	14 (19.7)	197 (42.5)		133 (40.9)	24 (39.3)	109 (41.3)	
Sometimes	52 (9.7)	5 (7.0)	47 (10.1)		29 (8.9)	4 (6.7)	25 (9.5)	
Where does child play when mother is bathing?								
Playing in water/bathing also	190 (35.5)	34 (47.9)	156 (33.6)	0.018*	120 (36.9)	29 (47.5)	91 (34.5)	0.100
Outside the water	123 (23.0)	18 (25.4)	105 (22.6)		79 (24.3)	15 (24.6)	64 (24.2)	
Playing in a water filled dish outside the river	222 (41.5)	19 (26.8)	203 (43.8)		126 (38.8)	17 (27.9)	109 (41.3)	
The usual source of water for bathing the child								
River	175 (32.7)	41 (57.8)	134 (28.9)	< 0.001*	110 (33.9)	21 (34.4)	89 (33.7)	0.698
Dam	6 (1.1)	0 (0)	6 (1.3)		6 (1.9)	0 (0)	6 (2.3)	
Well	56 (10.5)	10 (14.1)	46 (9.9)		35 (10.8)	7 (11.5)	28 (10.6)	
Protected well/borehole/tap	298 (55.7)	20 (28.2)	278 (59.9)		174 (53.5)	33 (54.1)	141 (53.4)	
Do you boil water for bathing the child?								
Yes	373 (69.7)	45 (63.4)	328 (70.7)	< 0.001*	216 (66.5)	40 (65.6)	176 (66.7)	0.176
No	34 (6.4)	12 (16.9)	22 (4.7)		25 (7.7)	8 (13.1)	17 (6.4)	
Sometimes	128 (23.9)	14 (19.7)	114 (24.6)		84 (25.9)	13 (21.3)	71 (26.9)	
Whether child aged ≤ 5 years assist in watering the garden								
Yes	136 (25.4)	31 (43.7)	105 (22.6)	< 0.001*	84 (25.9)	19 (31.2)	65 (24.6)	0.566
No	377 (70.5)	33 (46.5)	344 (744)		228 (70.2)	40 (65.6)	188 (71.2)	
Sometimes	22 (4.1)	7 (9.9)	15 (3.2)		13 (4.0)	2 (3.3)	11 (4.2)	
Presence of a toilet at home	426 (79.6)	59 (83.1)	367 (79.1)	0.435	260 (80.0)	49 (80.3)	211 (79.9)	0.943
Those using the toilet	454 (84.9)	66 (93.0)	388 (83.6)	0.041*	278 (85.5)	55 (90.2)	223 (84.5)	0.254
Source of drinking water								
River	40 (7.5)	11 (15.5)	29 (6.3)	< 0.001*	19 (5.9)	4 (6.6)	15 (5.7)	0.228
Protected well at home	127 (23.7)	14 (19.7)	113 (24.4)		86 (26.5)	19 (31.2)	67 (25.4)	
Borehole	337 (63.0)	36 (50.7)	301 (64.9)		202 (62.2)	32 (52.5)	170 (64.4)	
Garden well	31 (5.8)	10 (14.1)	21 (4.5)		18 (5.5)	6(9.8)	12 (4.6)	

^a^For the variables with a yes or no response, only the number (%) of the yes response are documented

^b^Children were not interviewed but they are described against their caregiver’s responses

^c^More than one response was considered

*Significance level of 0.05; **Borderline significance of 0.05

While 207 (63.7%) caregivers used borehole water for domestic purposes, its use was significantly higher in the uninfected caregivers (175, 66.3%) compared to those infected (32, 52.5%) (*χ*^2^ = 4.0977, *df* = 1, *P* = 0.043). Meanwhile, the use of river water for domestic purposes was significantly higher (*χ*^2^ = 15.2925, *df* = 1, *P* ≤ 0.001) in caregivers of infected children (27, 38.0%) compared to those of uninfected children (83, 17.9%). While 37 (52.1%) of the infected children’s caregivers used river water for bathing, the use of different bathing water sources among the caregivers of infected and uninfected children was significantly different (*χ*^2^ = 10.4170, *df* = 4, *P* = 0.034). Meanwhile, 41 (57.8%) of the infected children’s caregivers reported that they usually bath their children using river water and the difference in infection among children with varying sources of water for bathing was significantly different (*χ*^2^ = 28.7692, *df* = 3, *P* ≤ 0.001). For caregivers who took their children to the river for bathing, infection was significantly different (*χ*^2^ = 8.0110, *df* = 2, *P* = 0.018) among children who played or bathed in river water (34, 47.9%), children who played in a water-filled basin (19, 26.8%) and played outside of the water 18 (25.4%). There was a significantly higher number of infected children (60, 84%) for caregivers who took their children to the river for laundry activities compared to those who did not (11, 15.5%) (*χ*^2^ = 11.8896, *df* = 2, *P* = 0.003). Consequently, the number of children who were allowed to play in shallow water when the caregivers were performing laundry activities was significantly different between the infected and uninfected category (*χ*^2^ = 10.2804, *df* = 3, *P* = 0.016).

### Association of knowledge, perception and practices of caregivers with urogenital schistosomiasis infection

Table 6 describes the univariate and multivariate logistic regression analysis for the association of knowledge, perception and practices of caregivers with their urogenital schistosomiasis infection.

**Table 6:** Association of knowledge, perception and practices of caregivers with their schistosomiasis infection

Variable	Response	Univariate analysis			Multivariate analysis		
		OR	95% CI	*P*-value	Adj OR	95% CI	*P*-value
Perceptions							
School children are the high-risk group	Yes				1		
	No	0.16	0.3–1.2	0.123	0.4	0.2–0.9	0.025*
Adult men are the high-risk group	Yes	1			1		
	No	3.1	0.9–10.4	0.070	5.2	1.2–21.9	0.024*
Practices							
Those who use borehole water for domestic purposes	Yes	1			1		
	No	1.8	1.0–3.1	0.045*	2.2	1.1–4.3	0.020*
Source of water for bathing	Protected well	1			1		
	River	2.0	1.1–3.5	0.023*	3.0	1.4–6.4	0.004*
	Garden well	2.9	0.5–16.4	0.235	6.5	0.8–56.3	0.088
	Borehole	1.3	0.3–6.2	0.762	0.9	0.1–10.0	0.941
Time of bathing	Afternoon	1			1		
	Morning	0.9	0.2–4.8	0.949	0.9	0.1–6.9	0.934
	Late afternoon	1.9	0.9–4.1	0.094	2.4	1.0–5.7	0.055**
Use of a toilet for excreta disposal	Yes	1			1		
	No	0.6	0.2–1.5	0.259	0.2	0.1–0.9	0.037*

*Notes*: Reference group is marked as OR or Adj OR = 1. Univariate and multivariate logistic regression analysis of *S. haematobium* infection in caregivers in relation to sources of water for domestic use, sources of water for laundry, bathing and timing of bathing. In addition, the association of *S. haematobium* infection with toilet use and perceptions is also presented

*Significance association of 0.05; **Borderline significance of 0.05

*Abbreviations*: OR, odds ratio; 95% CI, 95% confidence interval; Adj OR, adjusted odds ratio

Among the caregivers, the univariate analysis showed that the risk of acquiring schistosomiasis was associated with the source of water that was being used for domestic purposes (OR: 1.8, 95% CI: 1.0–3.1) and bathing in river water (OR: 2.0, 95% CI: 1.1–3.5) only. After controlling for possible confounders, the multivariate logistic regression analysis showed that the risk of infection was high for caregivers who did not use borehole water for domestic purposes (AOR: 2.2, 95% CI: 1.1–4.3) compared to those who did. The risk was also high for caregivers using river water for bathing (AOR: 3.0, 95% CI: 1.4–6.4) compared to those who use protected well water. Caregivers who did not believe adult men are a high-risk group were at high odds of being infected. Meanwhile, caregivers who did not believe SAC are a high-risk group were 60% less likely to be infected (AOR: 0.4, 95% CI: 0.2–0.9) compared to those who believed SAC are a high-risk group.

### Association of knowledge, perception and practices of caregivers with schistosomiasis infection in PSAC

Table 7 describes the univariate and multivariate logistic regression analysis for association of knowledge, perception, and practices of caregivers with their PSAC’s urogenital schistosomiasis infections.

**Table 7:** Association of knowledge, perception and practices of caregivers with PSAC’s schistosomiasis infection

Variable	Response	Univariate analysis			Multivariate analysis		
		OR	95% CI	*P*-value	Adj OR	95% CI	*P*-value
Caregiver’s infection status	Not infected	1			1		
	Infected	3.9	1.9–7.7	< 0.001	3.9	1.7–8.6	0.001
Knowledge							
Caregiver indicates they are currently infected	No	1					
	Yes	4.2	2.1–8.2	< 0.001			
	I don’t know	1.5	0.7–3.1	0.316			
Perceptions							
Which group is most likely infected	School-aged children						
	Yes	1			1		
	No	1.3	0.8–2.4	0.326	2.6	1.1–5.9	0.022
	Adult women						
	Yes	1					
	No	0.6	0.3–1.0	0.065			
Practices							
Source of water for domestic purpose	Borehole						
	Yes	1					
	No	2.0	1.2–3.4	0.005			
Source of water for bathing	Protected well	1					
	River	2.2	1.3–3.7	0.003			
	Dam	2.4	0.3–22.1	0.443			
	Garden well	3.8	0.7–20.6	0.119			
	Borehole	1.2	0.3–5.5	0.819			
Is the toilet used?	Yes	1			1		
	No	0.4	0.2–1.0	0.048	0.1	0.0–0.5	0.009

*Notes*: Reference group is marked as OR or Adj OR = 1. Univariate and multivariate logistic regression analysis of *S. haematobium* infection in PSAC in relation to sources of domestic water, gardening and bathing practices. In addition, the association of *S. haematobium* infection with toilet use and knowledge of current infection status by the caregiver is also presented

*Significance association of 0.05; **Borderline significance of 0.05

*Abbreviations*: OR, odds ratio; 95% CI, 95% confidence interval; Adj OR, adjusted odds ratio

Univariate analysis showed that knowledge of caregiver’s current infection status, use of borehole and river water for domestic purposes, use of river water by the caregiver for bathing, allowing the child to play in water when the caregiver is bathing in the river, and bathing the child in river water were all risk factors for schistosomiasis infection. The perceptions that SAC and adult women are high-risk groups for schistosomiasis were not significant with the univariate analysis. Multivariate analysis showed that the odds of being infected was higher for PSAC of caregivers who had schistosomiasis infection (AOR: 3.9, 95% CI: 1.7–8.6) and whose caregivers believed that SAC are a high-risk group.

## Discussion

The present study demonstrates that urogenital schistosomiasis is a significant public health problem in caregivers and their PSAC in Shamva District. This adds to the growing evidence regionally [18, 22, 28] on schistosomiasis epidemiology in PSAC and their caregivers and underlines the need to include them in current schistosomiasis treatment programmes. Moreover, the study has contributed to the scarce data demonstrating the KPP of caregivers as risk factors for schistosomiasis infection for themselves and their PSAC.

The difference in knowledge of disease symptoms was generally not significant between infected and uninfected caregivers in the area. Overall, the caregivers generally had a low knowledge of schistosomiasis prevention and control, which will enable sustained infection and transmission in such high-risk communities.

Women did not perceive themselves or their PSAC to be high-risk groups. This results in exposing PSAC, unknowingly, to infection through water contact activities such as allowing them to play or perform activities in the unprotected water while the caregiver is bathing or washing clothes. While most of the caregivers reported the use of toilets for excreta disposal, the few individuals who are infected and are practicing indiscriminate disposal of excreta puts the communities at risk of infection. Moreover, indiscriminate excreta disposal coupled with risky water contact activities reported here, such as bathing in the river or unprotected water sources, highly predispose individuals to schistosomiasis infection. Midzi et al. [29] had earlier reported indiscriminate disposal of excreta and poor water contact practices of SAC from endemic communities in Zimbabwe. Profiling the associations of such practices within an area assists in explaining spatial heterogeneities of disease prevalence [11, 12], in addition to identifying areas needing health education for behavioural change.

While overall disease prevalence was significantly different between caregivers and PSAC, it was higher in caregivers (18.8%) compared to the PSAC (13.3%), substantiating that schistosomiasis infection is a function of duration of exposure [30]. Caregivers have had or have more water contact exposure, thus are likely to have a higher prevalence compared to PSAC. These findings are similar to observations made in Zanzibar [28].

Regarding PSAC, the highest urogenital schistosomiasis prevalence was in the 5-year age group with no infections found in all the children less than one year old and only four (5.3%) children out of the 72 children aged one year were infected. These results corroborated findings reported in Nigeria [31], demonstrating an increase in prevalence of urogenital schistosomiasis infection with the age of the children. As the children grow, their independence also increases resulting in more repeated exposures to infections as they repeatedly play in open and often infective water sources. Limited safe water sources and lack of knowledge on prevention and control of the disease result in a high reliance on unprotected water sources in the study area. Thus, the observed early infections among PSAC less than three years of age will be due to early exposure to contaminated water bodies when they accompany their mothers for different water contact activities, as reported in other settings [18, 22, 28]. This early exposure is regardless of gender as *S. haematobium* infection was not significantly different between male and female PSAC.

Among all the communities, heavy intensity infections were low, but it could not be clarified why they were highest in Kaziro even though this area had a relatively low prevalence of infection. Identifying, targeting and profiling such individuals with high infection intensities is essential to understanding disease transmission characteristics for maximising control interventions [12]. Moreover, if left unattended, these heavy infections might result in poor growth and cognition in these children [32, 33] and also the development of female and male genital schistosomiasis with added pathological consequences [20].

In this study, the number of recruited caregivers was lower than the PSAC. While some caregivers had more than one PSAC under their care, some caregivers also sent their child along with their neighbour who also brought their own child or children to the urine collection point. Some caregivers ended up bringing three or more PSAC for screening. In the same context, PSAC might be exposed to infection while they are under the care of alternative carers or relatives who might also take them to the unprotected water bodies while performing their water contact activities. This partly explains why some caregivers were not infected yet their children were infected although age related acquired resistance to infection could not be ruled out [34].

The present prevalence recorded in Madziwa, Shamva District among the PSAC (13.3%) is lower than the one recorded in Murewa District, northeast Zimbabwe (~ 20%) in the same age group [16]. However, both Murewa and Shamva districts, according to the WHO classification [6], are high transmission areas having a prevalence of infection greater than 50% [25]. The difference in prevalence may be due to different socio-demographic factors, behavioural practices among the caregivers of the PSAC, in addition to difference in availability of water and sanitation facilities in these two districts; however, more studies are needed to identify the cause of these differences.

The prevalence of infection in caregivers recorded during this study (18.7%) is lower than the one previously recorded in the same area by Ndhlovu et al. [19] in 2007 (29% and above). However, as opposed to the previous study which was inclusive of all women aged 15–49 years in the area, our study only focused on caregivers of PSAC, possibly resulting in an underestimation of the true infection prevalence. Nevertheless, the schistosomiasis prevalence observed provides a starting point for considering large-scale screening of the adult populations so that appropriate intervention strategies can be implemented.

## Conclusions

According to the WHO schistosomiasis classification, women and PSAC in Madziwa area belong to the moderate risk category and mass chemotherapy once every two years is recommended to avert the associated morbidity for these specific age groups [35]. Knowledge, perceptions and water contact practices of caregivers are risk factors for schistosomiasis infection in caregivers and their PSAC. Thus, adult women in endemic communities should be included in the design, implementation and evaluation of schistosomiasis control programmes in view of their important role as caregivers, their educational role in raising the awareness of the risk of schistosomiasis infection in their children and as main house hold users of water from non-safe sources.

## Data Availability

The data supporting the conclusions of this article are included within the article. The datasets analysed during the present study are available from the corresponding author upon reasonable request.

## Abbreviations

KPP: knowledge, perceptions and practices
PSAC: preschool-aged children
SAC: school-aged children
WASH: water, sanitation and hygiene
WHA: World Health Assembly
WHO: World Health Organization
